# Reduced IgG titers against pertussis in rheumatoid arthritis: Evidence for a citrulline-biased immune response and medication effects

**DOI:** 10.1371/journal.pone.0217221

**Published:** 2019-05-28

**Authors:** Caitlyn L. Holmes, Chloe G. Peyton, Amy M. Bier, Tobias Z. Donlon, Fauzia Osman, Christie M. Bartels, Miriam A. Shelef

**Affiliations:** 1 Department of Medicine, University of Wisconsin-Madison, Madison, Wisconsin, United States of America; 2 Department of Pathology and Laboratory Medicine, University of Wisconsin-Madison, Madison, Wisconsin, United States of America; 3 William S. Middleton Memorial Veterans Hospital, Madison, Wisconsin, United States of America; CEA, FRANCE

## Abstract

**Background:**

The antibody response to pertussis vaccination in rheumatoid arthritis is unknown, a concerning omission given the relatively low efficacy of the pertussis vaccine, a rise in pertussis infections, and a general increased susceptibility to infection in rheumatoid arthritis. Additionally, the contributions from an intrinsically dysregulated immune system in rheumatoid arthritis and immune-suppressing medications to the response to pertussis vaccination is poorly defined. This study examines antibody titers against pertussis in vaccinated rheumatoid arthritis patients and controls as well as evaluates potential contributions from demographic factors, immune suppressing medications, and reactivity against citrullinated pertussis.

**Methods:**

Serum IgG titers against native and citrullinated pertussis and tetanus were quantified by enzyme-linked immunosorbent assay in rheumatoid arthritis subjects and controls who were vaccinated within 10 years. Titers were compared by t-test and percent immunity by Fisher’s exact test. Multivariable logistic regression was used to identify clinical factors that correlate with native pertussis titers.

**Results:**

Compared to controls, rheumatoid arthritis subjects had lower titers against pertussis, but not tetanus, and reduced immunity to pertussis. These results were even more prominent at 5–10 years post-vaccination, when rheumatoid arthritis patients had 50% lower titers than controls and 2.5x more rheumatoid arthritis subjects were not considered immune to pertussis. Multiple logistic regression demonstrated that female sex and methotrexate use, but not TNF inhibiting medications, correlated with reduced immunity to pertussis. Finally, rheumatoid arthritis patients had higher IgG titers against citrullinated pertussis than native pertussis.

**Conclusions:**

Pertussis titers are lower in vaccinated rheumatoid arthritis patients with evidence for contributions from female sex, a citrulline-biased immune response, and methotrexate use.

## Introduction

Patients with rheumatoid arthritis, a chronic progressive autoimmune disease with a lifetime risk of about 3% [1], are at increased risk for infection [2], but data are mixed regarding response to vaccination. Following influenza or pneumococcus immunization, which are both recommended for rheumatoid arthritis patients given overall efficacy [3, 4], rheumatoid arthritis patients have a normal response to some vaccine strains and serotypes and an impaired response to others [5–8], which may be improved by the use of adjuvant [9]. Also, patients with rheumatoid arthritis have similar antibody levels against tetanus compared to controls, but differences in antibody affinity and subclass [10]. Given the variability seen in the response of rheumatoid arthritis patients to different vaccines, it is necessary to separately assess the response to each vaccine. However, no studies have addressed the antibody response to the pertussis vaccine in rheumatoid arthritis.

*Bordetella pertussis* is a bacterial species that causes “whooping cough,” a severe respiratory infection characterized by violent and uncontrollable coughing associated with high rates of rib fractures and syncope in adults and apnea, pneumonia, and death in babies. An estimated 16 million cases of pertussis were reported globally in 2008 and incidence in the United States has been rising since 2002 [11, 12]. For adults in the United States, vaccination against pertussis is typically part of the Tdap (tetanus, diphtheria, and pertussis) vaccine, which is recommended to be administered every 10 years [13] since protection against pertussis from vaccination wanes after 4–12 years [14]. Patients with inflammatory bowel disease were recently shown to have reduced titers against pertussis [15]. Given the rise in the incidence of pertussis, the increased risk of infection in rheumatoid arthritis, and the reduced response of rheumatoid arthritis patients to some vaccines, it is important to determine if rheumatoid arthritis patients make a normal antibody response to pertussis vaccination.

The mechanism behind the altered response to some vaccines in rheumatoid arthritis is unclear. One possible mechanism is an inherently dysregulated immune system. People with rheumatoid arthritis generate autoantibodies against many different citrullinated proteins with overlapping specificity [16–19] and strong reactivity against citrulline itself [20], starting years prior to the diagnosis of rheumatoid arthritis [21]. This aberrant immune response extends to non-self antigens, since in rheumatoid arthritis, antibodies bind a citrullinated Epstein-Barr virus peptide more than the native peptide [22]. However, this citrulline-bias has never been evaluated in the context of vaccine response. Additionally, patients with rheumatoid arthritis take immune suppressing medications that could reduce the response to vaccination [6, 23–25]. Indeed, a brief discontinuation of methotrexate in rheumatoid arthritis patients can improve the response to vaccination against influenza [26]. The contributions of these mechanisms are unknown for the antibody response to pertussis vaccination in rheumatoid arthritis.

Here, we evaluate if antibody titers against pertussis in vaccinated rheumatoid arthritis patients are different than controls. We also examine demographics, immune suppressing medications, and reactivity against citrullinated pertussis to identify potential factors involved with the antibody response against pertussis in rheumatoid arthritis.

## Materials and methods

### Human subjects

Research was carried out and subjects gave written informed consent in compliance with the Declaration of Helsinki and as approved by the Institutional Review Board at the University of Wisconsin-Madison (#2015–0156). All clinical data and biologic samples were obtained from the University of Wisconsin (UW) Rheumatology Biorepository first described in [27]. The biorepository contains clinical data (obtained from the electronic medical record and subject self-report) and serum from subjects at least 18 years old receiving primary care and rheumatology care (for rheumatoid arthritis subjects) in an academic health system. Potential rheumatoid arthritis subjects were initially identified as individuals with two or more outpatient visits with rheumatoid arthritis associated ICD codes (ICD-9 codes 714.0–714.33, 714.9 or any ICD-10 code starting with M05, M06, or M08) within 24 months [28] or one visit and a positive anti-CCP (cyclic citrullinated peptide) antibody test. Rheumatoid arthritis diagnosis was confirmed based on manual review of the three most recent rheumatologist progress notes in the electronic medical record. Subjects were selected for this study if they had received a Tdap vaccine within 10 years of the blood collection for the biorepository. Rheumatoid arthritis subjects also had positive anti-CCP and rheumatoid factor tests with values twice the upper limit of normal. Since rituximab eliminates B cells preventing an antibody response, subjects using rituximab were excluded. Controls were matched by age and gender and excluded if they had any of the following diagnoses as determined by verbal screen or manual record review: systemic lupus erythematosus, Sjögren’s Syndrome, scleroderma, multiple sclerosis, type I diabetes, psoriasis or psoriatic arthritis, ankylosing spondylitis, reactive arthritis, ulcerative colitis, Crohn’s disease, cancer of the blood cells including leukemia or lymphoma.

For all subjects, the following variables were included as abstracted from the medical record for the time of serum collection unless otherwise noted: rheumatoid arthritis diagnosis, age, sex, smoking status and history, body mass index (BMI), Charlson comorbidity score [29], prescription of non-steroidal anti-inflammatory drugs (NSAIDs), and time since Tdap vaccination. For rheumatoid arthritis subjects, we also included age of rheumatoid arthritis diagnosis (self-reported since some subjects were diagnosed prior to inclusion in our electronic medical record), which was used to determine if a subject had rheumatoid arthritis at the time of vaccination (for subjects self-reporting rheumatoid arthritis diagnosis within six months of vaccination date, diagnosis date by the subject’s rheumatologist in the electronic medical record was used), and prescription of the following medications at the time of serum collection and vaccination as abstracted from the medical record: abatacept, hydroxychloroquine, leflunomide, methotrexate, sulfasalazine, and tumor necrosis factor (TNF) inhibitor (includes adalimumab, etanercept, and infliximab). No subjects were prescribed certolizumab or golimumab. Tofacitinib and tocilizumab were not included in the analysis since 4 and 0 subjects were prescribed this medication, respectively.

### Serum preparation

For the biorepository, blood was collected from subjects into serum separator tubes (Greiner Bio-One, Monroe, USA) and centrifuged at 1300x g for 10 minutes. Serum was transferred to a fresh tube and centrifuged at 2000x g for 5 minutes. The supernatant was then aliquoted and stored at -80°C.

### Enzyme linked immunosorbent assay (ELISA)

Pertussis and tetanus IgG titers were measured using *Bordetella pertussis* and Tetanus toxoid IgG ELISA kits according to the manufacturer’s instructions (Immuno-Biological Laboratories, Inc., Minneapolis, USA). Per manufacturer’s instruction, immunity to pertussis was defined as a pertussis IgG titer higher than 20 U/mL. For citrullination ELISAs, the precoated wells of pertussis and tetanus toxoid IgG ELISA kits were incubated with citrullination buffer (100mM Tris-HCl pH7.5, 1mM DTT, and 5mM CaCl_2_) alone or with buffer and 0.01μg/mL peptidylarginine deiminase (PAD) 4 and 0.01μg/mL PAD2 overnight at 37°C similar to previously [30]. Wells were washed three times before proceeding with the ELISA per manufacturer’s instruction. As a negative control, non-precoated 96 well plates (EIA/RIA Plate High Binding, Costar, Corning, USA) were exposed to buffer alone or buffer with PAD enzymes as above and used in ELISA to detect IgG in sera that binds to PAD enzymes. To assess citrullination efficiency, the pertussis and tetanus precoated wells exposed to buffer alone or buffer with PAD enzymes were washed three times before proceeding with the ELISA with these modifications: mouse anti-citrulline IgM (clone F95, EMD Millipore, Darmstadt, Germany) diluted 1:200 as primary antibody and anti-mouse IgM-HRP (SouthernBiotech, Birmingham, USA) diluted 1:5000 as secondary antibody.

### Statistical analysis

Mean pertussis titers were compared between rheumatoid arthritis subjects and controls using a t-test and antibody levels against native versus citrullinated antigen by paired t-test. Comparison for proportional immunity between groups was measured with a Fisher’s exact test. To determine which clinical factors correlated with pertussis titer, univariate and multiple logistic regression analyses were performed. The multiple logistic regression models compared patients to median pertussis values across the entire cohort as well as proportions determined to be clinically immune to pertussis for odds ratio (OR) comparisons. For all statistical tests, p<0.05 was considered significant. Statistical analysis was performed using Stata version 14 (StataCorp LP, College Station, USA) and Prism (GraphPad Software, San Diego, USA).

## Results

Ninety-eight rheumatoid arthritis patients and seventy-seven controls who received the Tdap vaccine within 10 years of blood collection were selected from the UW Rheumatology Biorepository. Controls and rheumatoid arthritis patients were similar with regards to age, sex, race/ethnicity, smoking status, BMI, time since vaccination, and age at vaccination (S1 Table). Time since vaccination demonstrated a trend towards being slightly shorter for rheumatoid arthritis subjects as compared to controls, by an average of 9.6 months (p = 0.06). Also, consistent with the one point given for a rheumatoid arthritis diagnosis in the scoring system, the Charlson comorbidity score was higher in rheumatoid arthritis subjects.

**Fig 1:**
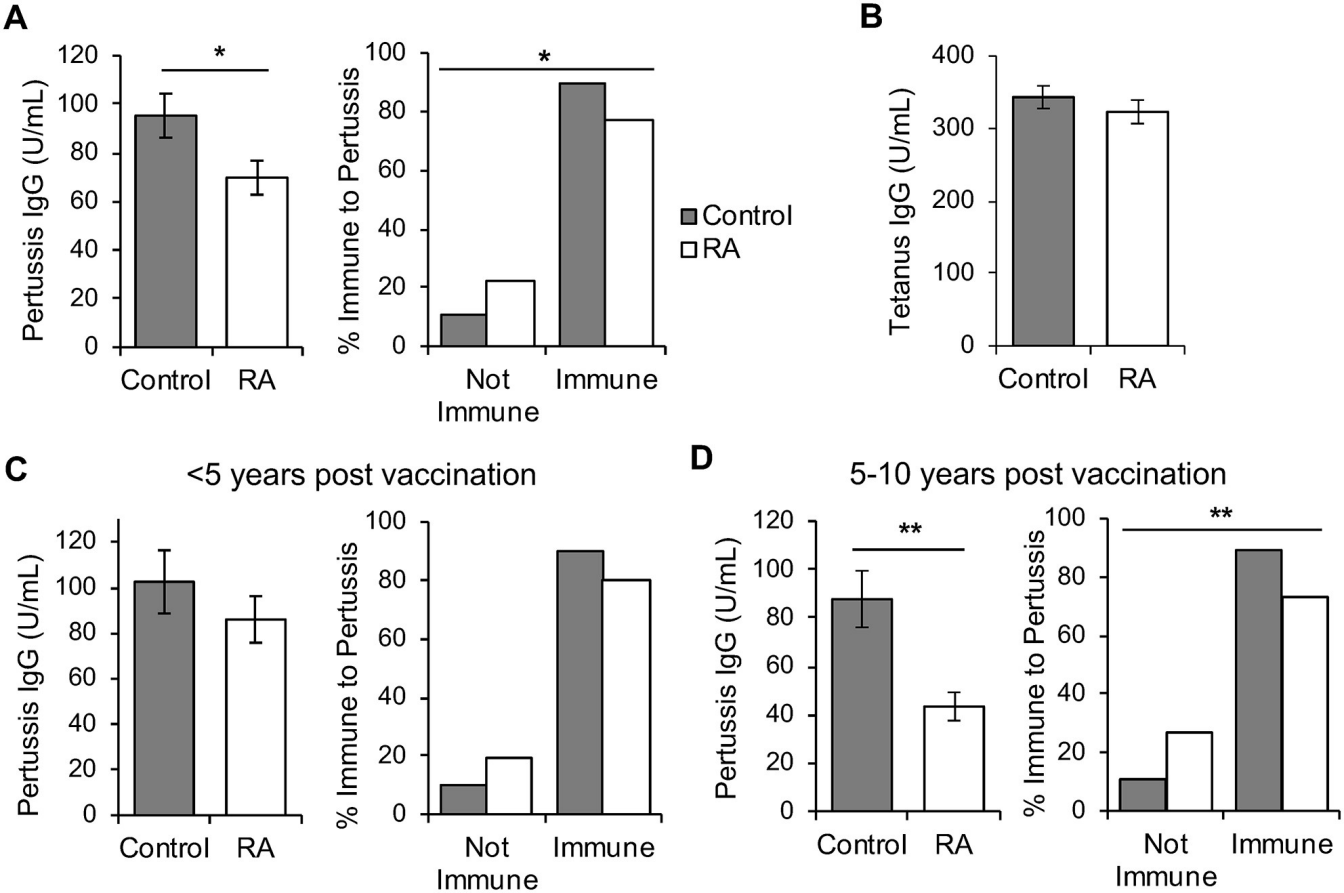
Pertussis titers are lower in rheumatoid arthritis subjects than controls especially 5–10 years post-vaccination. Sera from rheumatoid arthritis (RA) patients and controls were subjected to ELISA to detect IgG titers against pertussis (A) with averages and SEM graphed (left) as well as the percent of each group considered immune to pertussis (right) (controls n = 77, RA n = 98). (B) Sera were also subjected to ELISA to detect IgG titers against tetanus with average and SEM graphed (controls n = 77, RA n = 98). Subjects were divided into Tdap vaccination <5 years (C) or 5–10 years (D) prior to serum collection with average pertussis IgG titers and SEM graphed (left) as well as the percent of subjects considered immune to pertussis (right). For subjects <5 years post-vaccination: control n = 40, RA n = 61. For subjects 5–10 years post-vaccination: control n = 37, RA n = 37. In all panels, *p<0.05, **p<0.01.

Sera from control and rheumatoid arthritis subjects were subjected to ELISA to detect IgG against pertussis and tetanus. As shown in Fig 1A (left panel), rheumatoid arthritis subjects had lower pertussis IgG titers compared to controls. Moreover, more than twice as many rheumatoid arthritis patients were considered not immune to pertussis than controls (22% versus 10%, p = 0.03, Fig 1A, right panel). As expected [10], no significant difference in tetanus IgG titers was observed between rheumatoid arthritis subjects and controls (Fig 1B).

Since pertussis immunity wanes after 4–12 years [14], we divided subjects by Tdap vaccination less than 5 years prior to serum collection or 5–10 years prior to serum collection and compared differences in pertussis IgG titers for rheumatoid arthritis versus control subjects. Rheumatoid arthritis subjects had only a trend towards reduced titers and reduced rates of immunity compared to controls <5 years after vaccination (Fig 1C). However, 5–10 years post-vaccination, rheumatoid arthritis subjects had about 50% lower titers compared to controls and 2.5 times more rheumatoid arthritis subjects were not immune (Fig 1D). Taken together, these data suggest that patients with rheumatoid arthritis have lower titers against pertussis and reduced rates of immunity against pertussis, particularly 5–10 years after vaccination.

To determine if clinical variables, such as use of immune suppressing medications, correlated with median pertussis titers, univariate and multiple logistic regression analyses were performed. When modeled for controls and rheumatoid arthritis subjects, a diagnosis of rheumatoid arthritis, female sex, and time since vaccination predicted lower than median pertussis titers in both univariate and multivariable analyses (Table 1). Obesity predicted higher than median titers in a univariate model, but this finding was not significant in the multivariable model (Table 1). When rheumatoid arthritis subjects were evaluated separately, female sex was again predictive of lower than median pertussis titers (Table 2). Also, methotrexate use at the time of serum collection predicted lower than median pertussis titers in the univariate, but not the multivariable model (Table 2). In contrast, leflunomide use predicted higher than median pertussis titers in the univariate analysis only (Table 2). Time since vaccination again predicted lower than median pertussis titers and, interestingly, being diagnosed with rheumatoid arthritis at the time of vaccination (as opposed to not yet being diagnosed with rheumatoid arthritis at the time of vaccination) predicted greater than median pertussis titers in the univariate analysis with similar trends for both in the multivariable analysis.

**Table 1:** Predictors of greater than median pertussis titer in rheumatoid arthritis and control subjects (n = 98 rheumatoid arthritis and 77 controls).

	Univariate			Multivariable		
	OR	95% CI	p	OR	95% CI	p
Rheumatoid Arthritis	**0.48**	**(0.26, 0.89)**	**0.02**	**0.29**	**(0.13, 0.63)**	**0.002**
Age	1.00	(0.97, 1.02)	0.73	0.97	(0.93, 1.01)	0.12
Sex: Female	**0.38**	**(0.20, 0.75)**	**0.005**	**0.34**	**(0.16, 0.71)**	**0.005**
Smoking Status (Never)	Ref.			Ref.		
Current	1.53	(0.53, 4.41)	0.43	1.57	(0.49, 5.05)	0.45
Former	1.48	(0.77, 2.87)	0.24	1.34	(0.62, 2.91)	0.45
BMI (Normal)	Ref.			Ref.		
Overweight	1.49	(0.65, 3.44)	0.35	1.34	(0.53, 3.36)	0.53
Obese	**2.31**	**(1.09, 4.93)**	**0.03**	1.94	(0.85, 4.46)	0.12
Charlson Comorbidity Score	1.04	(0.90, 1.20)	0.57	1.19	(0.92, 1.54)	0.19
NSAIDs (n = 77)	0.89	(0.49, 1.61)	0.70	0.92	(0.46, 1.85)	0.81
Time since Vaccination	**0.84**	**(0.75, 0.95)**	**0.007**	**0.83**	**(0.72, 0.95)**	**0.006**

**Table 2:** Predictors of greater than median pertussis titer in rheumatoid arthritis (RA) (n = 98).

	Univariate			Multivariable		
	OR	95% CI	p	OR	95% CI	p
Age	1.01	(0.97, 1.04)	0.68	0.99	(0.93, 1.06)	0.85
Sex: Female	**0.28**	**(0.12, 0.67)**	**0.004**	**0.26**	**(0.09, 0.75)**	**0.01**
Smoking Status (Never)	Ref.			Ref.		
Current	1.71	(0.44, 6.62)	0.43	1.84	(0.36, 9.25)	0.46
Former	1.61	(0.66, 3.90)	0.29	2.02	(0.55, 7.45)	0.29
BMI (Normal)	Ref.			Ref.		
Overweight	2.35	(0.75, 7.36)	0.14	3.20	(0.82, 12.50)	0.10
Obese	2.48	(0.87, 7.08)	0.09	2.38	(0.66, 8.58)	0.19
Charlson Comorbidity Score	1.09	(0.90, 1.31)	0.40	0.85	(0.56, 1.30)	0.46
NSAIDs (n = 54)	1.27	(0.57, 2.86)	0.56	1.09	(0.38, 3.14)	0.87
Time since Vaccination	**0.80**	**(0.67, 0.96)**	**0.01**	0.81	(0.65, 1.02)	0.08
RA at Vaccination (n = 70)	**3.67**	**(1.33, 10.14)**	**0.01**	3.51	(0.90, 13.72)	0.07
Abatacept (n = 7)	0.53	(0.10, 2.89)	0.47	0.34	(0.05, 2.44)	0.29
Hydroxychloroquine (n = 23)	0.86	(0.33, 2.24)	0.76	0.84	(0.21, 3.34)	0.80
Leflunomide (n = 20)	**3.32**	**(1.19, 9.28)**	**0.02**	3.50	(0.78, 15.61)	0.10
Methotrexate (n = 53)	**0.41**	**(0.18, 0.94)**	**0.04**	0.71	(0.21, 2.43)	0.59
Sulfasalazine (n = 7)	1.95	(0.41, 9.21)	0.40	4.54	(0.41, 50.25)	0.22
TNF inhibitor (n = 35) ^a^	1.53	(0.67, 3.54)	0.32	1.16	(0.34, 3.95)	0.82

^a^ For TNF inhibitor users: 17 were prescribed methotrexate, 4 leflunomide, 5 hydroxychloroquine, and 1 sulfasalazine with some subjects taking more than one of these medications.

We then performed univariate and multiple logistic regression analyses to identify clinical variables that correlate with immunity to pertussis according to cut-offs provided by the ELISA kit manufacturer. When modeled for controls and rheumatoid arthritis subjects combined, a diagnosis of rheumatoid arthritis and female sex predicted lower immunity to pertussis (Table 3), similar to the analysis for median pertussis titers (Table 1). Longer time since vaccination showed a trend towards predicting reduced immunity (Table 3), whereas time since vaccination clearly predicted lower than median titers (Table 1) perhaps due to titers falling over time, but not necessarily below the level required for immunity in this analysis. When modeled for rheumatoid arthritis subjects alone, female sex again predicted lower immunity (Table 4). Additionally, in multivariable analysis, methotrexate use at the time of serum collection predicted significantly lower immunity to pertussis (Table 4), similar to our univariate findings for median pertussis titers (Table 2). Of note, medications prescribed at the time of vaccination were also analyzed in place of medications prescribed at the time of serum collection for subjects diagnosed with rheumatoid arthritis at the time of vaccination, but no medication significantly correlated with either greater than median pertussis titer or pertussis immunity when prescribed at the time of vaccination (S2 and S3 Tables).

**Table 3:** Predictors of pertussis immunity in rheumatoid arthritis and control subjects (n = 98 rheumatoid arthritis and 77 controls).

	Univariate			Multivariable		
	OR	95% CI	p	OR	95% CI	p
Rheumatoid Arthritis	**0.40**	**(0.17, 0.96)**	**0.04**	**0.30**	**(0.11, 0.81)**	**0.02**
Age	0.99	(0.96, 1.03)	0.74	0.99	(0.94, 1.03)	0.55
Sex: Female	**0.30**	**(0.10, 0.91)**	**0.03**	**0.27**	**(0.08, 0.85)**	**0.03**
Smoking Status (Never)	Ref.			Ref.		
Current	3.75	(0.47, 30.02)	0.21	3.93	(0.47, 33.19)	0.21
Former	1.44	(0.59, 3.50)	0.42	1.32	(0.49, 3.51)	0.59
BMI (Normal)	Ref.			Ref.		
Overweight	1.03	(0.38, 2.84)	0.95	0.88	(0.30, 2.59)	0.82
Obese	1.73	(0.66, 4.57)	0.27	1.39	(0.50, 3.87)	0.53
Charlson Comorbidity Score	0.97	(0.81, 1.16)	0.74	1.04	(0.76, 1.42)	0.81
NSAIDs (n = 77)	1.03	(0.47, 2.28)	0.94	1.02	(0.43, 2.43)	0.97
Time since Vaccination	0.91	(0.78, 1.07)	0.26	0.90	(0.76, 1.06)	0.21

**Table 4:** Predictors of pertussis immunity in rheumatoid arthritis (RA) (n = 98).

	Univariate			Multivariable		
	OR	95% CI	p	OR	95% CI	p
Age	0.99	(0.95, 1.03)	0.69	0.99	(0.93, 1.06)	0.81
Sex: Female	0.34	(0.11, 1.11)	0.07	**0.23**	**(0.06, 0.91)**	**0.04**
Smoking Status (Never)	Ref.			Ref.		
Current	2.93	(0.34, 25.21)	0.33	3.91	(0.36, 2.71)	0.26
Former	1.12	(0.40, 3.14)	0.84	1.09	(0.31, 3.92)	0.89
BMI (Normal)	Ref.			Ref.		
Overweight	1.35	(0.39, 4.72)	0.64	1.55	(0.33, 7.29)	0.58
Obese	1.43	(0.46, 4.46)	0.54	1.48	(0.38, 5.69)	0.57
Charlson Comorbidity Score	1.00	(0.80, 1.26)	0.97	0.88	(0.61, 1.27)	0.49
NSAIDs (n = 54)	1.03	(0.40, 2.67)	0.95	0.79	(0.26, 2.37)	0.67
Time since Vaccination	0.89	(0.73, 1.08)	0.24	0.85	(0.66, 1.09)	0.21
RA at Vaccination (n = 70)	1.22	(0.44, 3.42)	0.70	1.02	(0.25, 4.13)	0.97
Abatacept (n = 7)	0.35	(0.07, 1.71)	0.20	0.24	(0.03, 1.87)	0.17
Hydroxychloroquine (n = 23)	1.06	(0.34, 3.26)	0.93	0.81	(0.20, 3.22)	0.76
Leflunomide (n = 20)	0.84	(0.27, 2.63)	0.76	0.21	(0.03, 1.29)	0.09
Methotrexate (n = 53)	0.47	(0.17, 1.27)	0.14	**0.15**	**(0.03, 0.82)**	**0.03**
Sulfasalazine (n = 7) ^a^	-	-	-	-	-	-
TNF inhibitor (n = 35) ^b^	1.25	(0.45, 3.44)	0.67	0.46	(0.11, 1.98)	0.30

^a^ All subjects using sulfasalazine were immune to pertussis limiting calculations.

^b^ For TNF inhibitor users: 17 were prescribed methotrexate, 4 leflunomide, 5 hydroxychloroquine, and 1 sulfasalazine with some subjects taking more than one of these medications.

**Fig 2:**
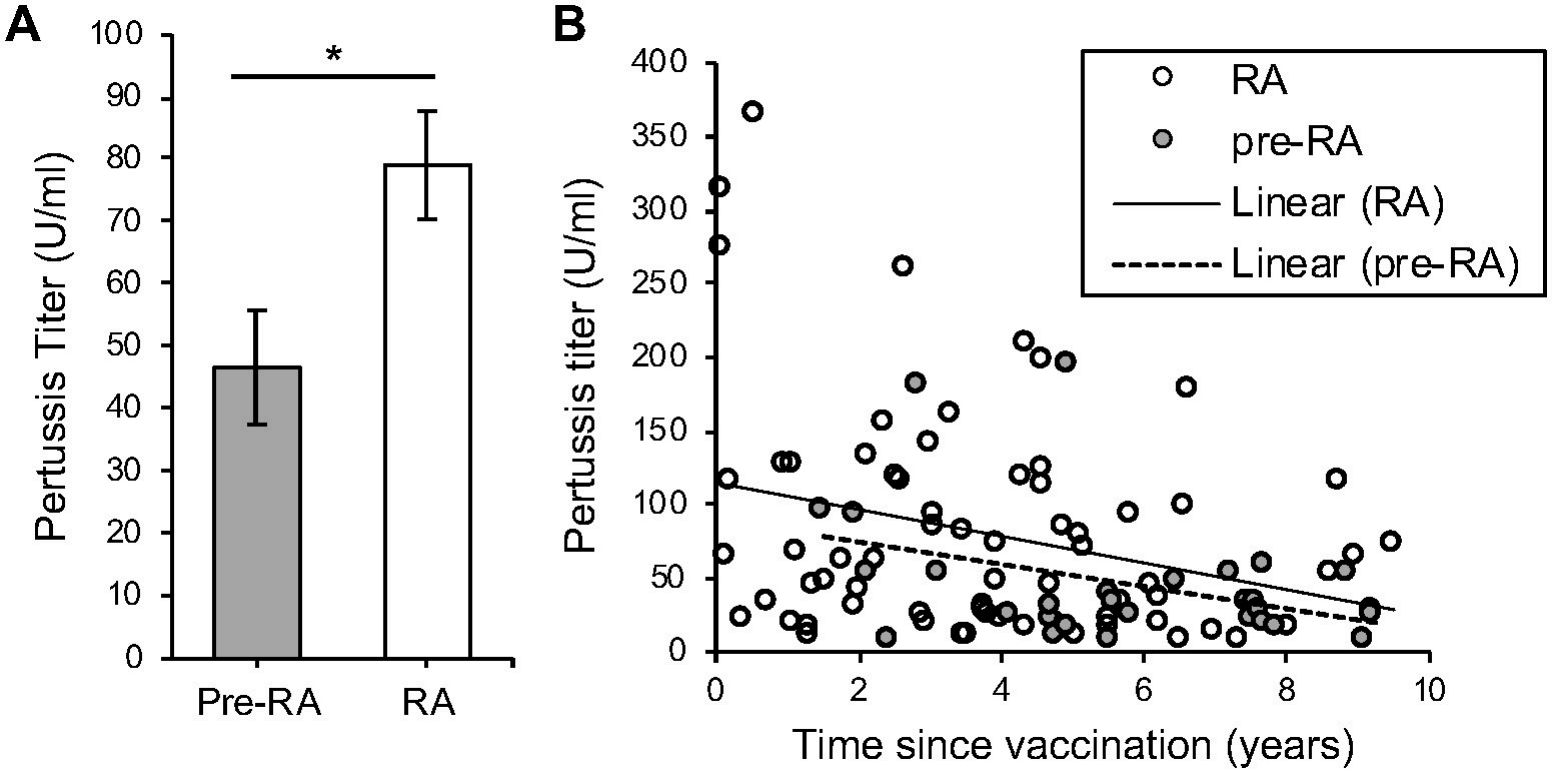
Lower pertussis titers in subjects not yet diagnosed with rheumatoid arthritis as compared to diagnosed with rheumatoid arthritis at the time of Tdap vaccination. A. Pertussis IgG titers were compared between subjects not yet diagnosed with rheumatoid arthritis (pre-RA) versus diagnosed with rheumatoid arthritis (RA) at the time of vaccination by t-test with averages and SEM graphed. B. Pertussis IgG titers and time since vaccination were graphed for pre-RA and RA groups with linear trendlines calculated. For all panels n = 28 pre-RA, n = 70 RA, *p<0.05.

We next determined if there was evidence of an inherently dysregulated immune system affecting the antibody response to pertussis vaccination in rheumatoid arthritis. We were intrigued by the univariate finding that a diagnosis of rheumatoid arthritis, as opposed to not yet being diagnosed, at the time of Tdap vaccination predicted greater than median pertussis titers (Table 2). The subjects without a diagnosis of rheumatoid arthritis at vaccination would be diagnosed 0.1 to 6.6 years later. Given known delays in diagnosis [31] and the common presence of anti-citrullinated protein antibodies (ACPAs) a decade prior to diagnosis [21], the subjects without a rheumatoid arthritis diagnosis very likely had undiagnosed rheumatoid arthritis or preclinical rheumatoid arthritis at the time of vaccination. However, none of these subjects were receiving immune suppression, providing an opportunity to evaluate a role for immune dysregulation in vaccine response. Thus, we compared pertussis titers in subjects with versus without a diagnosis of rheumatoid arthritis at the time of vaccination and found significantly lower titers in subjects not yet diagnosed with rheumatoid arthritis (Fig 2A). The undiagnosed subjects had a longer time since vaccination than subjects diagnosed with rheumatoid arthritis at the time of vaccination (average 1.8 years). Since a longer time since vaccination is also associated with lower pertussis titers (Fig 1 and Table 2), we divided subjects by diagnosis or no diagnosis of rheumatoid arthritis at the time of vaccination and, for each group, plotted pertussis titer versus time since vaccination. As shown in Fig 2B, there is a trend towards reduced titers over time in both groups as well as lower titers in undiagnosed subjects suggesting that both time since vaccination as well as untreated, pre-diagnosed rheumatoid arthritis correlate with lower pertussis titers.

**Fig 3:**
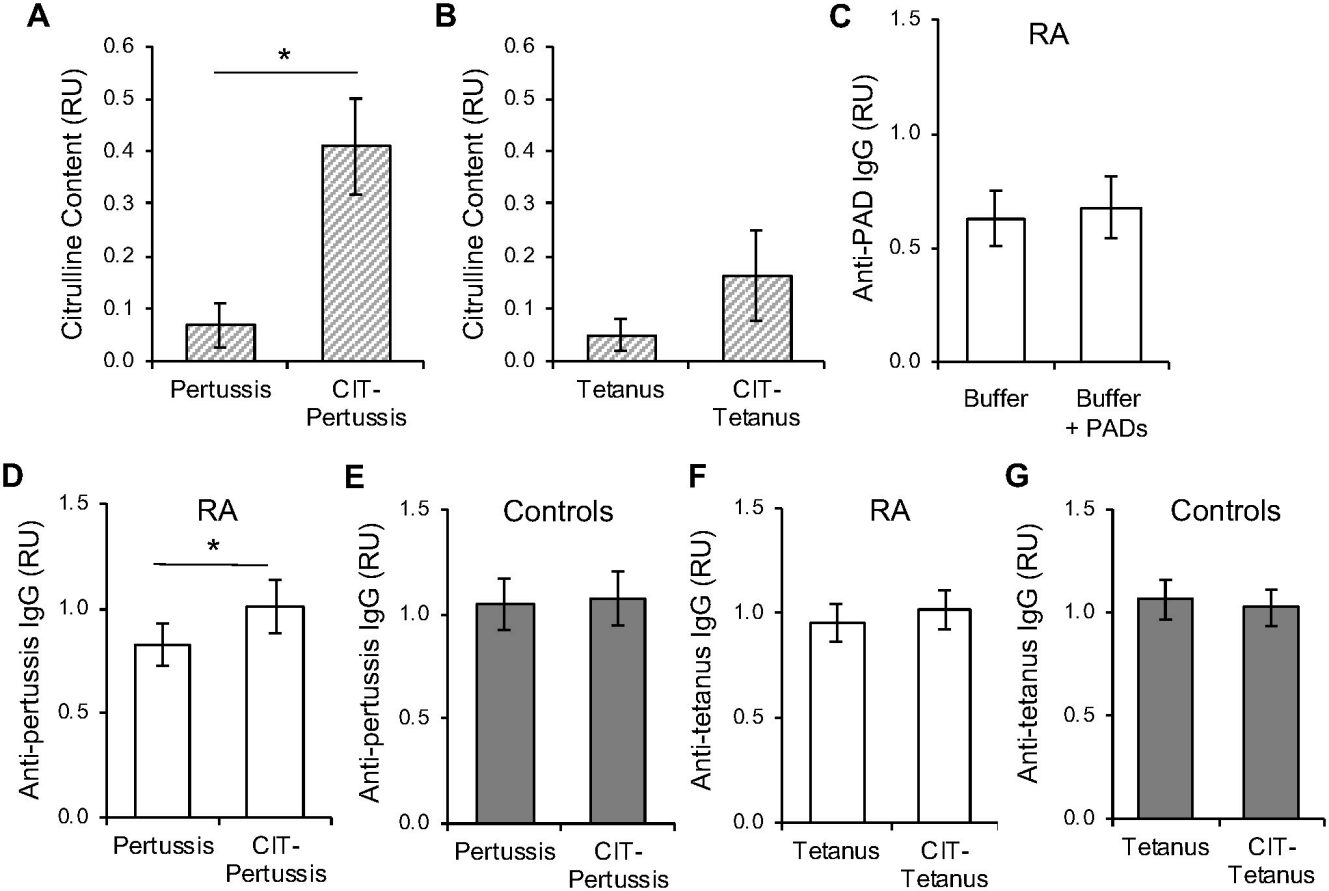
Rheumatoid arthritis patients have higher IgG binding to citrullinated than native pertussis. Following citrullination of pertussis-coated (A) and tetanus toxoid-coated (B) wells, the extent of citrullination was quantified using an anti-citrulline antibody with the relative units (RU) compared for untreated versus citrullinated (CIT) wells. Averages with SEM are graphed (n = 5, *p<0.05). Sera from rheumatoid arthritis (RA) patients (C, D, F) and controls (E, G) were subjected to ELISA to detect IgG binding against PAD enzymes (C), native and citrullinated (CIT) pertussis (D, E), or native and potentially citrullinated tetanus (F, G) with averages and SEM graphed. For panels C-G, control n = 30, RA n = 31, *p<0.05.

Given these findings suggesting that immune dysregulation might contribute to lower pertussis titers in rheumatoid arthritis, we then hypothesized that rheumatoid arthritis patients would have antibodies that bind citrullinated pertussis, potentially reducing the normal immune response against native pertussis. To test this hypothesis, we treated the pertussis- and tetanus-coated ELISA plates with PAD2 and PAD4 and quantified citrullination by ELISA. As shown in Fig 3A and 3B, pertussis was efficiently citrullinated, but tetanus toxoid was not, likely due to the detoxification of tetanus toxin with formaldehyde to generate tetanus toxoid, a process which modifies arginines [32, 33]. We then demonstrated a lack of detectable binding of rheumatoid arthritis sera to the amount of PAD enzyme used to citrullinate (Fig 3C) and repeated our ELISAs to detect antibodies against citrullinated and native pertussis. Rheumatoid arthritis sera, and not control sera, had higher antibody binding to citrullinated compared to native pertussis (Fig 3D and 3E). Although we were unable to efficiently citrullinate tetanus toxoid *in vitro* and thus suspect that tetanus toxoid is also not citrullinated *in vivo*, we did see a trend towards increased citrullination and, thus, we determined if there was increased binding to the PAD-treated tetanus toxoid. As shown in Fig 3F and 3G, no increased binding was seen for rheumatoid arthritis or control sera against potentially citrullinated tetanus. Together, these data suggest that pertussis can be citrullinated and antibodies in rheumatoid arthritis bind citrullinated pertussis more than native pertussis, whereas tetanus toxoid is resistant to citrullination and thus is not likely to be a target of ACPAs.

## Discussion

In this report, we have demonstrated that pertussis IgG titers are significantly lower in rheumatoid arthritis subjects compared to controls. Moreover, the percent of rheumatoid arthritis subjects considered immune to pertussis is two-fold lower than controls. Both of these findings were even more prominent in subjects who received the pertussis vaccine 5–10 years prior to serum collection. These results could suggest that hundreds of thousands of rheumatoid arthritis patients in the United States alone may be susceptible to pertussis infection, despite receiving the Tdap vaccine according to national guidelines. Further, our findings, combined with the extremely low vaccination rates against pertussis in rheumatoid arthritis patients in Germany [34], could suggest that as the numbers of pertussis infections rise in general, pertussis could become a significant problem for rheumatoid arthritis patients.

Limitations of our study include its retrospective design and that it does not examine infection rates in rheumatoid arthritis. Additionally, while our results reflect immune status according to the cut-offs of a commercial assay, no laboratory correlate of definitive pertussis protection is known. It will be important for future studies to examine rates of pertussis infection in patients with rheumatoid arthritis, particularly since we detected antibodies that react with citrullinated pertussis, which may or may not be protective. Moreover, prospective studies could determine if more frequent or higher dose vaccination would improve the antibody response to pertussis in rheumatoid arthritis.

We also provide evidence for potential mechanisms for these reduced titers, which are important for understanding how rheumatoid arthritis patients respond to immunization. One possible mechanism for reduced pertussis IgG titers is the use of specific immune suppressing medications. In multivariable analysis, methotrexate use at the time of serum collection significantly correlated with lower pertussis immunity in rheumatoid arthritis (Table 4) with a similar finding of predicting less than median pertussis titers in univariate analysis (Table 2). These findings are similar to results seen for several studies evaluating methotrexate use and pneumococcal and influenza vaccines [6, 23, 24], although another study did not identify a correlation between methotrexate use and reduced vaccine response [8]. Additionally, a temporary pause in methotrexate usage at the time of influenza vaccination was shown to improve the response [26]. In contrast, we did not observe a correlation between methotrexate use at the time of vaccination and reduced response to pertussis (S2 and S3 Tables) potentially due to sample size, the retrospective nature of our study, or differences between the antibody response to pertussis versus influenza vaccines. Although the mechanism is not fully understood, methotrexate reduces T cell activity [35, 36]. Since acellular pertussis vaccines likely require T cells to establish humoral memory [37, 38], the depletion of T cells by methotrexate may contribute to the lower pertussis IgG titers in subjects taking this medication.

Interestingly, despite the high level of immune suppression attributed to TNF inhibiting medications, we did not see a correlation between TNF inhibitor use and reduced pertussis immunity or lower pertussis titers. This finding contrasts with a report that pertussis titers are lower in patients with inflammatory bowel disease using TNF inhibitors as compared to those using thiopurine [15]. For influenza and pneumonia vaccines, some studies report a correlation between TNF inhibiting medications and reduced titers, some studies report no reduction in titers related to TNF inhibitor use, and some studies suggest that reduced titers could be due to co-administration of additional immunomodulating agents with the TNF inhibiting medication [8, 24, 25, 39–44], as is the case in many of our subjects. The reason for this variability has not been thoroughly investigated. One possibility is that there may be differences among TNF inhibitors. For example, infliximab [41] and golimumab [25] are associated with reduced response to influenza and pneumococcal vaccines, whereas in separate studies, adalimumab, certolizumab, and etanercept use correlate with a normal antibody response to these vaccines [42–44]. Consistent with this theory, in our study, 34 of our 35 TNF inhibitor users were prescribed etanercept or adalimumab whereas in the inflammatory bowel disease study [15], 40% of the TNF inhibitor users were prescribed infliximab (F Caldera and M Hayney, University of Wisconsin-Madison, personal communication).

We also demonstrated that female sex significantly correlated with lower pertussis immunity and lower than median pertussis titers. This finding agrees with a study that found a correlation between reduced seropositivity for pertussis toxin and female sex in Hungary [45]. Sex differences in vaccine response occur for a variety of antigens although no clear pattern has been identified [46, 47]. Females have a greater response to influenza, hepatitis A and B, smallpox, and Brucella whereas males show greater response to pneumococcal polysaccharide and yellow fever vaccines [46, 48–54]. Both sexes respond similarly to the measles, mumps, and rubella (MMR) vaccine [55]. It remains unknown if sex differences in vaccine response are due to hormones, epigenetics, environment, microbiome, or cultural influences [47]. Future studies are needed to better characterize the role of sex in vaccine response and to optimize vaccine schedules based on gender and risk profiles.

Finally, our data suggest that an intrinsically dysregulated immune system may contribute to reduced pertussis titers in vaccinated rheumatoid arthritis subjects. We show that subjects vaccinated shortly prior to a diagnosis of rheumatoid arthritis (a disease phase associated with ACPAs as well as increased cytokines and chemokines [21, 56], but not immune suppressing medications) have lower pertussis titers than subjects diagnosed with rheumatoid arthritis at the time of vaccination. Moreover, we demonstrate that rheumatoid arthritis patients, but not controls, have greater binding to citrullinated than native pertussis, providing the first evidence that citrulline reactivity in rheumatoid arthritis extends to vaccine response. How antibodies against citrullinated pertussis develop and potentially contribute to reduced antibodies to native pertussis is not known. Vaccine adjuvants can attract neutrophils and induce neutrophil extracellular trap (NET) formation at the injection site [57]. Citrullinating PAD enzymes are released during NETosis and are present on NETs [58]. Thus, pertussis at the injection site could become citrullinated by PADs released from NETs. Alternatively, antigen processing can generate citrullinated peptides [59]. Rheumatoid arthritis patients may then preferentially generate antibodies against citrullinated as opposed to native pertussis antigens. Alternatively, given the cross-reactive repertoire of ACPAs in rheumatoid arthritis [16–20], pre-existing ACPAs could cross-react with pertussis citrullinated by NETs leading to clearance and reduced antigen availability for the generation of an immune response. Further work is needed to fully reveal this and other potential mechanisms that may alter the immune response to pertussis in rheumatoid arthritis.

Finally, in contrast to our pertussis results, we found that rheumatoid arthritis patients had normal titers against tetanus, as previously reported [10]. It is possible that immune suppressing medications do not sufficiently impair the immune system to reduce the antibody response to the highly effective tetanus vaccine, but do reduce the response to the less efficacious pertussis vaccine. Additionally, there may be inherent differences in the immune response against pertussis as compared to tetanus in rheumatoid arthritis. In support of this theory, we found increased IgG binding to citrullinated pertussis, whereas tetanus toxoid could not be efficiently citrullinated *in vitro* and thus is unlikely to be citrullinated *in vivo*, making the development of anti-citrullinated tetanus antibodies or cross-reactivity of ACPAs with tetanus extremely unlikely. Future studies are needed to examine differences in the immune response to vaccination against these different pathogens in rheumatoid arthritis.

## Conclusions

We have shown that vaccinated rheumatoid arthritis patients have lower titers against pertussis than controls with a potential role for female sex, methotrexate, and a citrulline-biased immune response. Our findings have potential clinical importance since they are the first to identify lower pertussis titers in rheumatoid arthritis, suggesting that these patients could be more susceptible to pertussis infection and might benefit from more frequent vaccination. Additionally, we provide the first evidence that a citrulline-biased immune system may complicate the response to immunization in rheumatoid arthritis, providing a novel mechanism for abnormal vaccine response.

## Supporting information

S1 Table A comparison of demographic and clinical characteristics between rheumatoid arthritis and control subjects. (DOCX)

S2 Table Predictors of greater than median pertussis titer in subjects diagnosed with rheumatoid arthritis at the time of vaccination including medications taken at the time of vaccination (n = 70). (DOCX)

S3 Table Predictors of pertussis immunity in subjects diagnosed with rheumatoid arthritis at the time of vaccination including medications taken at the time of vaccination (n = 70). (DOCX)
