# Supplementary material for: Reduced IgG titers against pertussis in rheumatoid arthritis: Evidence for a citrulline-biased immune response and medication effects
Source: PLoS One. 2019 May 28;14(5):e0217221. doi: 10.1371/journal.pone.0217221 (PMC6538243; doi:10.1371/journal.pone.0217221)
Supplement: S1 Table — (DOCX) [file pone.0217221.s001.docx]

| **Supplementary Table 1.** Characteristics of Rheumatoid Arthritis and Control Subjects | | | |
| --- | --- | --- | --- |
|  | Rheumatoid Arthritis | Control | p |
|  | (n=98) | (n=77) |  |
| Age, mean years (range) | 60 (32-85) | 59 (29-87) | 0.44 |
| Sex, female (%) | 64 (65.3) | 58 (75.3) | 0.15 |
| Race/Ethnicity, number (%) |  |  |  |
| White | 78 (79.6) | 66 (85.7) | 0.08 |
| Black | 3 (3.1) | 3 (3.9) |  |
| Hispanic | 8 (8.2) | 0 (0.0) |  |
| Native American | 8 (8.2) | 5 (6.5) |  |
| Asian | 1 (1.0) | 3 (3.9) |  |
| Pacific Islander | 0 (0.0) | 0 (0.0) |  |
| Smoking Status, number (%) |  |  |  |
| Current smoker | 10 (10.2) | 6 (7.8) | 0.80 |
| Former smoker | 31 (31.6) | 23 (29.9) |  |
| Never smoked | 57 (58.2) | 48 (62.3) |  |
| Body Mass Index, number (%) |  |  |  |
| Normal | 26 (26.5) | 17 (22.1) | 0.71 |
| Overweight | 28 (28.6) | 21 (27.3) |  |
| Obese | 44 (44.9) | 39 (50.7) |  |
| **Charlson Comorbidity Score, mean (SE)** | **3.5 (0.2)** | **2.1 (0.2)** | **<0.0001** |
| Time since Vaccination, mean years (SE) | 4.4 (0.3) | 5.2 (0.3) | 0.06 |
| Age at Vaccination, mean years (SE) | 55.6 (1.2) | 53.5 (1.5) | 0.25 |
