# Supplementary material for: Reduced IgG titers against pertussis in rheumatoid arthritis: Evidence for a citrulline-biased immune response and medication effects
Source: PLoS One. 2019 May 28;14(5):e0217221. doi: 10.1371/journal.pone.0217221 (PMC6538243; doi:10.1371/journal.pone.0217221)
Supplement: S2 Table — (DOCX) [file pone.0217221.s002.docx]

| **Supplementary Table 2.** Predictors of greater than median pertussis titer in subjects diagnosed with rheumatoid arthritis at the time of vaccination including medications taken at the time of vaccination (n=70) | | | | | | | |
| --- | --- | --- | --- | --- | --- | --- | --- |
|  | Univariate | | |  | Multivariable | | |
|  | OR | 95% CI | p |  | OR | 95% CI | p |
| Age | 0.98 | (0.94, 1.02) | 0.36 |  | 0.94 | (0.86, 1.04) | 0.23 |
| Sex: Female | **0.31** | **(0.11, 0.88)** | **0.03** |  | **0.22** | **(0.06, 0.90)** | **0.03** |
| Smoking Status (Never) | Ref. |  |  |  | Ref. |  |  |
| Current | 1.54 | (0.31, 7.79) | 0.60 |  | 1.73 | (0.24, 12.66) | 0.59 |
| Former | 1.39 | (0.49, 3.93) | 0.54 |  | 2.42 | (0.52, 11.34) | 0.26 |
| BMI (Normal) | Ref. |  |  |  | Ref. |  |  |
| Overweight | 2.33 | (0.62, 8.82) | 0.21 |  | 1.94 | (0.38, 9.92) | 0.43 |
| Obese | **3.89** | **(1.18, 12.84)** | **0.03** |  | 5.24 | (1.00, 27.40) | 0.05 |
| Charlson Comorbidity Score | 1.05 | (0.84, 1.31) | 0.66 |  | 1.14 | (0.59, 2.18) | 0.70 |
| NSAIDs (n=37) | 1.41 | (0.55, 3.62) | 0.47 |  | 0.67 | (0.18, 2.55) | 0.56 |
| Time since Vaccination | 0.88 | (0.72, 1.08) | 0.21 |  | 0.86 | (0.64, 1.16) | 0.34 |
| Abatacept (n=4)* | - | - | - |  | - | - | - |
| Hydroxychloroquine (n=14) | 1.43 | (0.44, 4.67) | 0.55 |  | 0.57 | (0.08, 4.09) | 0.57 |
| Leflunomide (n=12) | 2.30 | (0.62, 8.48) | 0.21 |  | 1.40 | (0.21, 9.49) | 0.73 |
| Methotrexate (n=26) | 0.61 | (0.23, 1.62) | 0.32 |  | 0.68 | (0.20, 2.30) | 0.54 |
| Sulfasalazine (n=5) | 4.39 | (0.46, 41.04) | 0.20 |  | 6.69 | (0.31, 146.29) | 0.23 |
| TNF inhibitor (n=28)** | 1.27 | (0.49, 3.31) | 0.63 |  | 0.68 | (0.16, 2.84) | 0.60 |
| *All subjects taking abatacept had lower than median pertussis titers. **For TNF inhibitor users: 11 were prescribed methotrexate, 3 leflunomide, 4 hydroxychloroquine, and 1 sulfasalazine with some subjects taking more than one of these medications. | | | | | | | |
