# Supplementary material for: Reduced IgG titers against pertussis in rheumatoid arthritis: Evidence for a citrulline-biased immune response and medication effects
Source: PLoS One. 2019 May 28;14(5):e0217221. doi: 10.1371/journal.pone.0217221 (PMC6538243; doi:10.1371/journal.pone.0217221)
Supplement: S3 Table — (DOCX) [file pone.0217221.s003.docx]

| **Supplementary Table 3.** Predictors of pertussis immunity in subjects diagnosed with rheumatoid arthritis at the time of vaccination including medications taken at the time of vaccination (n=70) | | | | | | | |
| --- | --- | --- | --- | --- | --- | --- | --- |
|  | Univariate | | |  | Multivariable | | |
|  | OR | 95% CI | p |  | OR | 95% CI | p |
| Age | 0.97 | (0.92, 1.03) | 0.35 |  | 1.01 | (0.92, 1.12) | 0.83 |
| Sex: Female | 0.59 | (0.17, 2.09) | 0.41 |  | 0.36 | (0.06, 2.03) | 0.25 |
| Smoking Status (Never) | Ref. |  |  |  | Ref. |  |  |
| Current* | - | - | - |  | - | - | - |
| Former | 1.10 | (0.32, 3.74) | 0.88 |  | 1.79 | (0.29, 11.01) | 0.53 |
| BMI (Normal) | Ref. |  |  |  | Ref. |  |  |
| Overweight | 1.50 | (0.35, 6.50) | 0.59 |  | 0.56 | (0.08, 3.96) | 0.56 |
| Obese | 2.31 | (0.60, 8.94) | 0.22 |  | 7.39 | (0.94, 58.26) | 0.06 |
| Charlson Comorbidity Score | 0.95 | (0.74, 1.22) | 0.67 |  | 0.82 | (0.44, 1.56) | 0.55 |
| NSAIDs (n=37) | 0.98 | (0.31, 3.06) | 0.97 |  | 0.46 | (0.10, 2.06) | 0.31 |
| Time since Vaccination | 0.89 | (0.70, 1.12) | 0.31 |  | 0.72 | (0.50, 1.04) | 0.08 |
| Abatacept (n=4) | 0.25 | (0.03, 1.91) | 0.18 |  | 0.03 | (0.001, 1.01) | 0.05 |
| Hydroxychloroquine (n=14) | 1.00 | (0.24, 4.17) | 1.00 |  | 0.15 | (0.02, 1.22) | 0.08 |
| Leflunomide (n=12) | 0.78 | (0.18, 3.35) | 0.74 |  | 0.10 | (0.01, 1.23) | 0.07 |
| Methotrexate (n=26) | 1.24 | (0.37, 4.12) | 0.73 |  | 1.31 | (0.27, 6.30) | 0.73 |
| Sulfasalazine (n=5)** | - | - | - |  | - | - | - |
| TNF inhibitor (n=28)*** | 1.44 | (0.43, 4.77) | 0.55 |  | 0.20 | (0.03, 1.67) | 0.14 |
| *All current smokers were immune to pertussis. **All subjects taking sulfasalazine were immune to pertussis. ***For TNF inhibitor users: 11 were prescribed methotrexate, 3 leflunomide, 4 hydroxychloroquine, and 1 sulfasalazine with some subjects taking more than one of these medications. | | | | | | | |
